# Development of an Educational Intervention and Patient-Family Facing Materials for Deprescribing in Home Hospice

**DOI:** 10.1093/geroni/igaa057.702

**Published:** 2020-12-16

**Authors:** Jennifer Tjia, Mardie Clayton, Vennesa Duodu, Geraldine Puerto, Lynley Rappaport, Susan DeSanto-Madeya

**Affiliations:** 1 UMass Medical School, Worcester, Massachusetts, United States; 2 University of Utah, Salt Lake City, Utah, United States; 3 Epidemiology, Worcester, Massachusetts, United States; 4 Boston College, West Roxbury, Massachusetts, United States

## Abstract

Deprescribing is emerging as a clinical intervention to optimize quality of life, improve patient safety, and reduce burden for older adults with serious illness and their caregivers. Few resources are available to educate and engage clinicians, older adults and their families in discussions about deprescribing. This presentation describes the development of educational intervention materials, including clinician, patient and family-facing materials, about medication management and deprescribing for seriously ill older adults in home hospice. An environmental scan of the existing deprescribing resources was conducted; a state-of-the-art educational program for hospice deprescribing was located and used as the basis for an innovative clinician-facing educational intervention. A stakeholder panel of 2 hospice administrators, 3 nurses, 2 physicians, 2 pharmacists, and 2 former family caregivers, drawn from 2 geographically diverse hospice agencies, reviewed the content of the educational program and made recommendations for additional content. Iterative rounds of development and feedback resulted in: (1) a 3-part, 1 hour, clinician educational program with CE credits for nurses that presents a standardized deprescribing approach that aligns medication prescribing with the goals of patients and caregivers, and (2) a patient/family caregiver medication management notebook for intervention group participants (addressing management of common symptoms, medications in hospice, and deprescribing) and an adapted version for attention control participants. A professional designer created thematic coherence for all materials that were well received by stakeholder panelists and hospice staff. Ultimately, educational materials will support efforts to standardize deprescribing approaches that aim to optimize end-of-life care outcomes for patients and family caregivers.

